# Risk factors associated with the recent cholera outbreak in Yemen: a case-control study

**DOI:** 10.4178/epih.e2019015

**Published:** 2019-04-21

**Authors:** Fekri Dureab, Albrecht Jahn, Johannes Krisam, Asma Dureab, Omer Zain, Sameh Al-Awlaqi, Olaf Müller

**Affiliations:** 1 The Modern Social Association, Aden, Yemen; 2 Heidelberg Institute of Global Health, Heidelberg University School of Medicine, Heidelberg, Germany; 3 Institute of Medical Biometry and Informatics, Heidelberg University School of Medicine, Heidelberg, Germany; 4 Health and Education Association for Development (SAWT), Aden, Yemen; 5 Community Medicine Department, Faculty of Medicine, University of Aden, Aden, Yemen; 6 Institute of Public Health, Jagiellonian University Medical College, Krakow, Poland

**Keywords:** Cholera, Case-control studies, Risk factors, Conflict, Khat, Yemen

## Abstract

**OBJECTIVES:**

The cholera outbreak in Yemen has become the largest in the recent history of cholera records, having reached more than 1.4 million cases since it started in late 2016. This study aimed to identify risk factors for cholera in this outbreak.

**METHODS:**

A case-control study was conducted in Aden in 2018 to investigate risk factors for cholera in this still-ongoing outbreak. In total, 59 cholera cases and 118 community controls were studied.

**RESULTS:**

The following risk factors were associated with being a cholera case in the bivariate analysis: a history of travelling and having had visitors from outside Aden Province; eating outside the house; not washing fruit, vegetables, and khat (a local herbal stimulant) before consumption; using common-source water; and not using chlorine or soap in the household. In the multivariate analysis, not washing khat and the use of common-source water remained significant risk factors for being a cholera case.

**CONCLUSIONS:**

Behavioural factors and unsafe water appear to be the major risk factors in the recent cholera outbreak in Yemen. In order to reduce the risk of cholera, hygiene practices for washing khat and vegetables and the use and accessibility of safe drinking water should be promoted at the community level.

## INTRODUCTION

Cholera continues to be a significant public health problem in many developing countries. It manifests as an acute watery diarrhoeal disease, and it is mainly transmitted through the faecal-oral route by contaminated water or food. The disease is caused by the bacterium *Vibrio cholerae*. It is considered to be laboratory-confirmed when *V. cholerae* O1 or O139 is detected in any patient’s diarrhoea or vomitus. The first cholera pandemic began in 1817, and the current (seventh) pandemic started in 1961in Indonesia and is caused by the El Tor biotype. After starting in Indonesia, it spread to Asia, Africa, Europe, the Middle East, and Latin America [1].

Yemen is situated in southwest Asia on the Arabian Peninsula. The country has a diverse topography, with mountainous areas in the north, desert in the east, and a coastal landscape in the south and the west. The country is divided administratively into 23 governorates (provinces) and 333 districts. The population of Yemen is more than 30 million as of 2017 [2]. Yemen is the most impoverished country in the Arabian Peninsula and among the poorest worldwide, with a gross domestic product (GDP) of US$ 1,106 [3]. The country is furthermore burdened by low literacy rates, poor governance, a high prevalence of poverty, frequent food insecurity, and widespread malnutrition [4].

The emergence of the armed conflict in March 2015 in Yemen has exacerbated the already critical economic, political, and humanitarian situation that had prevailed for more than a decade. Approximately 4 years into the conflict, the country’s GDP has contracted by more than 42%, and the humanitarian situation has been further aggravated by increased fighting, population displacement, the lack of basic commodities and services, and an accompanying overall breakdown of public and social services [5].

The occurrence of epidemics, especially cholera, is known to be an obvious sign of the disruption of basic services [6]. Several cholera outbreaks have occurred during the last 10 years in Yemen; there were 3 smaller outbreaks in 2009, 2010 and 2011, with case fatality rates (CFRs) of 5.5%, 1.3%, and 0.4%, respectively [7-9]. The most recent outbreak started in October 2016, with 1,423,700 suspected cases and 4,510 laboratory-confirmed cases reported from 306 of 333 districts through January 2019; these cases have resulted in 2,767 deaths, corresponding to a CFR of 0.2% [10].

The war in Yemen has left 18.8 million people in need of assistance or protection, including the need for access to safe drinking water and sanitation, and an estimated 14.8 million people have no or limited access to basic healthcare. Moreover, there is a chronic shortage of medical supplies, and about half of the health facilities are no longer functional [11]. Public health facilities have been severely weakened by the escalating conflict, which has been compounded by the financial crisis of the Ministry of Public Health and Population (MoPHP). The lack of an operational budget has resulted in further disruption of many healthcare services and poses a major threat to Yemen’s public health [12]. The conflict has led to fragmentation of the health system, with two independent ministries for health operational in each region of the country (the rebels are seizing the north, while the legitimate government controls the southern region). This fragmentation has negatively impacted the health system and the delivery of essential health services [13].

The cholera outbreak in Yemen is the largest in the recent history of this disease. The magnitude of this outbreak can probably be explained by the overall breakdown of public services, including hygiene and sanitation, associated with the war in Yemen. However, evidence on specific risk factors is needed to guide an appropriate public health response. This study aimed to identify such risk factors for the recent large cholera outbreak in Yemen.

Khat (*Catha edulis*) is a flowering plant that grows in the horn of Africa and in Yemen. It is socially accepted in Yemen, and its green leaves are regularly chewed by the majority of people and used as a cognitive stimulant. The amphetamine-like alkaloid cathinone in khat is known to cause excitement, loss of appetite, and euphoria [14]. As there have been rumours that using khat could be associated with the cholera outbreak, this study included the use of khat as a potential risk factor.

## MATERIALS AND METHODS

### Study design and procedures

**Figure 1.**
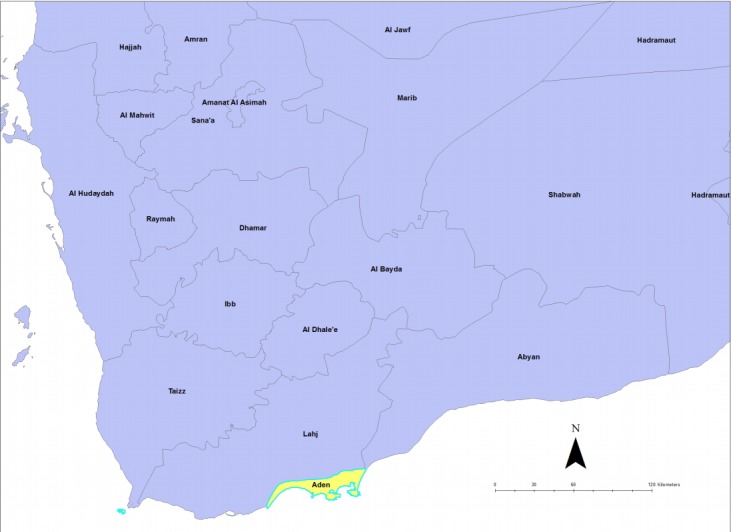
Map of Aden.

We reviewed the cholera outbreak line list of the MoPHP surveillance department in Aden, Yemen, from January 1 until March 31, 2018. The department is part of the health authorities of the Aden Province (Figure 1), and collects data on communicable diseases. The variables recorded in the line list were name, age, sex, residence, date of onset of diarrhoea, date of treatment or admission to a health facility, and outcome status.

This case-control study was conducted from April 1 to 30, 2018. A case was defined as any person suffering from watery diarrhoea between January 1 and March 31, 2018, whose illness was confirmed as cholera based on a laboratory analysis (isolation of *V. cholerae* serotypes 01 or 0139 in stool culture). Further inclusion criteria were being a resident of Aden and age ≥2 years because cholera is not common in children younger than 2 years [15]. The controls were defined as members of the same community with an absence of diarrhoea between January 1 and March 31, 2018. Two controls were matched to each case by sex and residence (district) from 7 out of the 8 districts in Aden Province (Table 1). The 2 controls were randomly selected from the 10 households to the right and to the left of the household of the case, respectively. If there was more than 1 person of the same sex as the case in a selected control household, the one closest to the age of the case was selected.

We calculated a minimum sample size of 177 that included 59 cases and 118 controls using the Power and Sample Size Programme. The assumptions made for the sample size calculation included a 25% exposure rate among controls, a power of 90% for the study, 95% confidence intervals (CIs), and a desired odds ratio (OR) to be detected of 3.

A standardised questionnaire was used to collect data on events that occurred during the 10 days preceding the onset of diarrhoea. The questionnaire was adapted from the cholera investigation form developed by the National Institute for Communicable Diseases in South Africa [16]. The questionnaire was administered by trained field staff to cases and controls through face-to-face interviews. The questions focused on demographic and socioeconomic information and on possible cholera risk factors such as handwashing practices, water sources, treatment and storage of drinking water, sources of food, hygiene and sanitation practices, khat chewing, and attendance at ceremonies.

### Data analysis

Data were entered into Excel spreadsheets, and all data were checked and cleaned by the local field supervisor. Double-checking was done by the principal investigator (FD). Exposure-related variables were included in the logistic regression analysis used in the multivariate model if they displayed a p-value<0.05 in the bivariate analysis. We calculated the ORs and 95% CIs associated with the independent variables. Data were analysed using SPSS version 25.0 (IBM Corp., Armonk, NY, USA).

### Ethics statement

Formal permission to conduct this study was obtained from the MoPHP Ethical Committee in Aden, Yemen. All participants provided oral and written informed consent before the interview. For participants below 18 years of age, parents or guardians granted consent on their behalf and accompanied them during the interview. No identifiable personal data were associated with the reported results, as the questionnaires were coded by numbers only.

## RESULTS

### Demographic and socioeconomic characteristics of the study population

The whole study population consisted of 177 participants: 59 cases (33.3%) and 118 (66.7%) controls. The distribution of participants by district is presented in Table 2.

The demographic and socioeconomic characteristics of the study population are presented in Table 2. There was a slight female sex predominance in the participants. The mean age of cases was not very different from the mean age of controls (31.7 years vs. 35.8 years), but there were more children below 10 years among the cases than among the controls. Approximately 5% of the participants were internally displaced persons (IDPs). Almost half of the study population had a monthly income of less than YER 40,000 (less than US$ 100). Overall, the cases and controls were well matched for most characteristics.

### Cholera risk factors

Table 3 shows the distribution of potential risk factors for cholera by study group. Concerning travel history and contacts, we found that traveling to another governorate, having had a visitor from another city, or having had contact with a potential cholera case were significantly associated with being a case. Among risks within dietary behaviour, eating outside the household was significantly associated with being a case, as were eating raw vegetables and fruits and not washing them before eating. Chewing khat was not associated with being a case; however, not washing khat before use was significantly associated with being a case. With regard to water and sanitation, using indoor municipal tap water was significantly associated with being a control, while using common-source water was significantly associated with being a case. Moreover, using chlorine in the household was significantly associated with being a control, but the availability of soap in the toilets was not significantly associated with being a control.

Table 4 shows the non-adjusted and adjusted findings from the logistic regression analysis of factors associated with cholera cases. In the bivariate analysis, people who had a travel history to neighbouring provinces were 10 times more likely to be cases (OR, 10.83; 95% CI, 1.23 to 94.98), while those who had visitors from other governorates of Yemen prior to onset of disease were more than 8 times more likely to be cases (OR, 8.50; 95% CI, 0.92 to 77.92). Moreover, people who had contact with cholera patients over the 10 days prior to disease onset were 3 times more likely to be cases (OR, 3.36; 95% CI, 1.13 to 9.94).

Cases were less likely to use indoor municipal tap water as their main source of drinking water (OR, 0.38; 95% CI, 0.15 to 0.93) and to use chlorine to treat water in the household (OR, 0.22; 95% CI, 0.05 to 0.10). However, they were more likely to use common-source water as their primary source of drinking water (OR, 6.57; 95% CI, 1.28 to 33.61). Washing khat before chewing was significantly less prevalent in cases (OR, 0.14; 95% CI, 0.05 to 0.42). Eating outside the house was significantly associated with being a case (OR, 2.97; 95% CI, 1.13 to 7.79).

In the multivariate analysis, only the practices of not washing khat before chewing it (OR, 0.11; 95% CI, 0.04 to 0.36) and drinking from common-source water (OR, 7.67; 95% CI, 1.16 to 50.74) were significantly associated with being a cholera case.

## DISCUSSION

This study aimed to identify risk factors for the cholera outbreak in Yemen, based on a case-control study with 59 cases and 118 controls in Aden Province in southern Yemen. In the bivariate analysis, several factors were shown to be associated with being a cholera case: a history of travelling; having had visitors from outside the province; eating outside the house; not having washed fruits, vegetables, and khat before use; use of common-source water; and not using chlorine or soap in the household.

In the multivariate analysis, only not washing khat and using common-source water (private common borehole water, donated water tanks, and purchased water) remained significant factors, whereas a history of travelling and eating outside the house were not significantly associated with being a case. These results support findings from other studies that have identified similar risk factors [17-23].

People in Yemen regularly chew khat on a daily basis, and it has been proposed to be a potential risk factor for cholera because many people chew khat without washing it properly. The findings of this study support this hypothesis.

This study has a number of limitations. First, it was conducted in conditions of war and insecurity, which made the study logistics and data collection rather complicated. Second, the age distribution was not fully comparable between cases and controls, as there were more children aged 3-10 years among the cases than among the controls. Third, the confidence intervals of the ORs were often large, making interpretations difficult. Demographic variables (number of household members, economic status, IDPs, and occupation) were not tested as risk factors. Finally, the interviews were carried out some time after patients’ recovery (range, 15-90 days). Therefore, recall bias may have occurred.

In conclusion, this study supports the association of cholera outbreaks with a breakdown of social infrastructure and poor hygiene measures and practices, in particular safe water supply. The findings of this study confirm a number of known risk factors for cholera, such as travel history and food hygiene. Moreover, it was demonstrated that not washing khat before chewing is a specific risk factor for cholera in Aden.

## Figures and Tables

**Table 1.** Distribution of study participants by district in Aden Province, Yemen

Districts	Cases	Controls
Al-Bouriqa	2 (3.4)	4 (3.4)
Dar-sad	27 (45.8)	54 (45.8)
Alshaikh-Othman	5 (8.5)	10 (8.5)
Al-Mansoura	1 (1.7)	2 (1.7)
Al-Mualla	7 (11.9)	14 (11.9)
Al-Tawahi	3 (5.1)	6 (5.1)
Sirah	14 (23.7)	28 (23.7)
Total	59 (100)	118 (100)

Values are presented as number (%).

**Table 2.** Demographic and socioeconomic characteristics of the study population

Characteristics		Cases	Controls	p-value^1^
Sex	Male	26 (44.0)	52 (44.0)	0.56
	Female	33 (56.0)	66 (56.0)	
Age (yr)	2-10	4 (6.8)	1 (0.8)	<0.05
	11-20	16 (27.1)	20 (19.9)	
	21-30	13 (22.0)	28 (23.7)	
	31-40	6 (10.2)	25 (21.2)	
	41-50	9 (15.3)	23 (19.5)	
	>50	11 (18.6)	21|(17.8)	
No. of household members (n)	1-5	13 (24.5)	25 (26.6)	0.76
	6-10	29 (54.7)	54 (57.4)	
	11-19	11 (20.8)	15 (16.0)	
Economic status (USD)	<100	27 (47.4)	54 (50.5)	0.82
	100- 200	13 (22.8)	18 (16.8)	
	>200	3 (5.3)	7 (6.5)	
	Don’t know	14 (24.6)	28 (26.2)	
IDP	Yes	4 (6.8)	5 (4.2)	0.60
	No	55 (93.2)	112 (95.7)	
Occupation	No job	21 (46.7)	34 (40.0)	0.52
	Working	16 (35.6)	39 (45.9)	
	Student	8 (17.8)	12 (14.1)	

Values are presented as number (%). USD, US dollar; IDP, internally displaced person.

1 Chi-square test.

**Table 3.** Distribution of the potential risk factors

Variables	Cases	Controls	p-value^1^
Travel history to other provinces			0.02
Yes	5 (8.5)	1 (0.8)	
No	54 (91.5)	117 (99.2)	
Having had visitors from other provinces			0.02
Yes	4 (6.8)	1 (0.8)	
No	55 (93.2)	117 (99.2)	
History of contact with a cholera patient			0.02
Yes	9 (15.3)	6 (5.1)	
No	50 (84.7)	112 (94.9)	
History of attending any gathering			0.25
Yes	2 (3.4)	1 (0.8)	
No	57 (96.6)	117 (99.2)	
Eating raw vegetables and fruit every day			0.06
Yes	57 (96.6)	104 (88.1)	
No	2 (3.4)	14 (11.9)	
Washing vegetables and fruit before eating			0.10
Yes	47 (79.7)	97 (89.0)	
No	12 (20.3)	12 (11.0)	
Eating outside the house			0.02
Yes	11 (23.9)	9 (9.6)	
No	35 (76.1)	85 (90.4)	
Drinking or eating dairy products prior to onset of cholera			0.15
Yes	25 (43.1)	37 (32.2)	
No	33 (56.9)	78 (67.8)	
Chewing khat			0.63
Yes	30 (50.8)	55 (47.0)	
No	29 (49.2)	62 (53.0)	
Washing khat before eating			<0.001
Yes	5 (16.7)	33 (58.9)	
No	25 (83.3)	23 (41.1)	
Drinking from household tap water			0.03
Yes	47 (79.7)	104 (91.2)	
No	12 (20.3)	10 (8.8)	
Drinking from common-source municipal tap water			0.01
Yes	6 (10.2)	2 (1.7)	
No	53 (89.8)	116 (98.3)	
Drinking from private/well/borehole water			0.73
Yes	1 (1.7)	1 (0.8)	
No	58 (93.3)	117 (99.2)	
Using chlorine in the household			0.03
Yes	2 (3.4)	16 (13.6)	
No	57 (96.6)	106 (86.4)	
Flush toilet at home			0.20
Yes	59 (100)	116 (98.3)	
No	0 (0.0)	2 (1.7)	
Soap available at the toilet			0.06
Yes	52 (88.1)	113 (95.8)	
No	7 (11.9)	5 (4.2)	

Values are presented as number (%).

1 Chi-square test.

**Table 4.** Risk of cholera infection in Aden Province, Yemen using a logistic regression model

Variables	Univariate analysis	Multivariate analysis
	OR (95% CI)	aOR (95% Cl)
Travel history	10.83 (1.23, 94.98)	8.58 (0.91, 80.60)
Having visitors from other provinces	8.50 (0.92, 77.92)	5.78 (0.49, 67.42)
History of contact with cholera patient	3.36 (1.13, 9.94)	2.12 (0.55, 8.14)
Drinking from indoor municipal tap water	0.38 (0.15, 0.93)	1.50 (0.47, 4.84)
Drinking from common-source municipal tap water	6.57 (1.28, 33.61)	7.67 (1.16, 50.74)
Using chlorine in the household	0.22 (0.05, 0.10)	0.25 (0.05, 1.17)
Washing khat before chewing it	0.14 (0.05, 0.42)	0.11 (0.04, 0.36)
Eating outside the house	2.97 (1.13, 7.79)	2.95 (0.98, 8.86)

OR, odds ratio; CI, confidence interval; aOR, adjusted odds ratio.

## References

[1] Clemens JD, Nair GB, Ahmed T, Qadri F, Holmgren J (2017). Cholera. Lancet.

[2] Institute for Health Metrics and Evaluation Yemen. http://www.healthdata.org/yemen.

[3] World Bank (2017). GDP per capita (current US$). https://data.worldbank.org/indicator/NY.GDP.PCAP.CD?locations=YE.

[4] De Souza LR (2017). Correlates of child undernutrition in Yemen. Bdg: J Glob South.

[5] Dureab F, Shibib K, Al-Yousufi R, Jahn A (2018). Yemen: cholera outbreak and the ongoing armed conflict. J Infect Dev Ctries.

[6] European Commission (2017). European civil protection and humanitarian aid operations: Yemen. https://ec.europa.eu/echo/where/middle-east/yemen_en.

[7] World Health Organization (2010). Cholera, 2009. Wkly Epidemiol Rec.

[8] World Health Organization (2011). Cholera, 2010. Wkly Epidemiol Rec.

[9] World Health Organization (2012). Cholera, 2011. Wkly Epidemiol Rec.

[10] World Health Organization (2019). Electronic integrated disease early warning and response system, Yemen. Wkly Epidemiol Bull.

[11] UN Office for the Coordination of Humanitarian Affairs (2016). 2017 Humanitarian needs overview: Syrian Arab Republic. https://reliefweb.int/report/syrian-arab-republic/2017-humanitarian-needs-overview-syrian-arab-republic-enar.

[12] United Nations Office for the Coordination of Humanitarian Affairs (2018). Humanitarian response plan: Yemen. https://reliefweb.int/sites/reliefweb.int/files/resources/20180120_HRP_YEMEN_Final.pdf.

[13] Qirbi N, Ismail SA (2017). Health system functionality in a low-income country in the midst of conflict: the case of Yemen. Health Policy Plann.

[14] Wabe NT (2011). Chemistry, pharmacology, and toxicology of khat (catha edulis forsk): a review. Addict Health.

[15] Siddique AK, Ahmed S, Iqbal A, Sobhan A, Poddar G, Azim T (2011). Epidemiology of rotavirus and cholera in children aged less than five years in rural Bangladesh. J Health Popul Nutr.

[16] National Institute for Communicable Diseases (2018). Cholera preparedness: an update for Physicians, accident & emergency practitioners and laboratorians. http://www.nicd.ac.za/wp-content/uploads/2017/03/Cholera_preparedness_20180917.pdf.

[17] Rosewell A, Addy B, Komnapi L, Makanda F, Ropa B, Posanai E (2012). Cholera risk factors, Papua New Guinea, 2010. BMC Infect Dis.

[18] Kone-Coulibaly A, Tshimanga M, Shambira G, Gombe NT, Chadambuka A, Chonzi P (2010). Risk factors associated with cholera in Harare City, Zimbabwe, 2008. East Afr J Public Health.

[19] Siddiqui FJ, Bhutto NS, von Seidlein L, Khurram I, Rasool S, Ali M (2006). Consecutive outbreaks of Vibrio cholerae O139 and V. cholerae O1 cholera in a fishing village near Karachi, Pakistan. Trans R Soc Trop Med Hyg.

[20] Davies-Teye BB, Vanotoo L, Yabani JB, Kwakye-Maclean C (2014). Socio-economic factors associated with cholera outbreak in southern Ghana, 2012: a case-control study. Value Health.

[21] Oyugi EO, Boru W, Obonyo M, Githuku J, Onyango D, Wandeba A (2017). An outbreak of cholera in western Kenya, 2015: a case control study. Pan Afr Med J.

[22] Moradi G, Rasouli MA, Mohammadi P, Elahi E, Barati H (2016). A cholera outbreak in Alborz Province, Iran: a matched case-control study. Epidemiol Health.

[23] O’Connor KA, Cartwright E, Loharikar A, Routh J, Gaines J, Fouché MD (2011). Risk factors early in the 2010 cholera epidemic, Haiti. Emerg Infect Dis.

